# Natural Fibres as a Sustainable Reinforcement Constituent in Aligned Discontinuous Polymer Composites Produced by the HiPerDiF Method

**DOI:** 10.3390/ma14081885

**Published:** 2021-04-10

**Authors:** Ali Kandemir, Marco L. Longana, Tulio H. Panzera, Gilberto G. del Pino, Ian Hamerton, Stephen J. Eichhorn

**Affiliations:** 1 Department of Aerospace Engineering, Bristol Composites Institute, School of Civil, Aerospace, and Mechanical Engineering, University of Bristol, Queen’s Building, University Walk, Bristol BS8 1TR, UK; m.l.longana@bristol.ac.uk (M.L.L.); ian.hamerton@bristol.ac.uk (I.H.); s.j.eichhorn@bristol.ac.uk (S.J.E.); 2 Centre for Innovation and Technology in Composite Materials—CITeC, Department of Mechanical Engineering, Federal University of São João Del Rei-UFSJ, São João Del-Rei 36307-352, Brazil; panzera@ufsj.edu.br; 3 Departamento de Engenharia Mecânica, Universidade do Estado de Amazonas, Manaus 69050-030, Brazil; gpino@uea.edu.br

**Keywords:** aligned discontinuous fibre composites, flax fibres, mechanical properties, natural fibres, sustainable composites

## Abstract

Sustainable fibre reinforced polymer composites have drawn significant attention in many industrial sectors as a means for overcoming issues with end-of-life regulations and other environmental concerns. Plant based natural fibres are considered to be the most suitable reinforcement for sustainable composites since they are typically from renewable resources, are cheap, and are biodegradable. In this study, a number of plant based natural fibres-curaua, flax, and jute fibres-are used to reinforce epoxy, poly(lactic acid) (PLA), and polypropylene (PP) matrices to form aligned discontinuous natural fibre reinforced composites (ADNFRC). The novel HiPerDiF (high performance discontinuous fibre) method is used to produce high performance ADNFRC. The tensile mechanical, fracture, and physical (density, porosity, water absorption, and fibre volume fraction) properties of these composites are reported. In terms of stiffness, epoxy and PP ADNFRC exhibit similar properties, but epoxy ADNFRC shows increased strength compared to PP ADNFRC. It was found that PLA ADNFRC had the poorest mechanical performance of the composites tested, due principally to the limits of the polymer matrix. Moreover, curaua, flax (French origin), and jute fibres are found to be promising reinforcements owing to their mechanical performance in epoxy and PP ADNFRC. However, only flax fibre with desirable fibre length is considered to be the best reinforcement constituent for future sustainable ADNFRC studies in terms of mechanical performance and current availability on the market, particularly for the UK and EU.

## 1. Introduction

Fibre reinforced polymer matrix composites (FRP) are a vital class of lightweight engineering materials that display high specific stiffness and strength. Since they are lightweight, FRP are key materials for most engineering applications, e.g., aerospace, automotive, marine and wind energy [1,2,3,4]. However, the main constituents of FRP, the matrix and fibres, are difficult to recycle at end-of-life, and since they are predominantly petroleum-based non-renewable materials this prevents them being truly sustainable [5]. Therefore, the use of sustainably derived FRP is becoming an unavoidable option for petroleum-based components in structural engineering applications, due to growing public and industrial environmental awareness [6].

Most of the widely used synthetic reinforcements for FRP, such as carbon and glass fibres, are not renewable and the processes available to recycle them are not economically feasible [7,8]. The production of these fibres, especially carbon fibre, relies on limited sources, whereas natural fibres (NF) are abundantly available around the world in various forms [9]. NF are an ideal reinforcement constituent as being not only renewable and relatively cheap, they are also recyclable materials; in addition, NF can be produced sustainably and can simplify waste management processes [10]. Their wear performances in FRP, which can trigger energy efficiency and cost effectiveness during the processing stages, are better than those of their synthetic counterparts [11]. Even though NF have some drawbacks, such as low thermal stability and hydrophilic character, they have the potential to exhibit high performance in FRP. They are key alternatives of synthetic fibres (e.g., aramid, carbon, and glass) that do not share mentioned sustainable features of NF [12].

Most synthetic fibres can be produced in a well-aligned continuous format able to achieve high performance in FRP for engineering applications, whereas NF are limited by the characteristic dimensions of their source and can only be processed into relatively long, aligned, discontinuous fibres. However, to employ aligned continuous fibres to the manufacture of composites is labour intensive, and incurs high environmental impact and cost during production [13,14]. On the other hand, composites produced in this manner may exhibit high mechanical performance, even comparable to continuous fibre composites, when a high degree of alignment is achieved with fibres of lengths greater than the critical fibre length [15]. The discontinuities in the reinforcement improve their formability, facilitating defect-free manufacturing of structural parts with complex shapes [16]. Consequently, the cost and environmental impact of production can be reduced significantly. The HiPerDiF (high performance discontinuous fibre) method is a discontinuous FRP production technology, invented at the University of Bristol [17], that has been shown to manufacture FRP materials rapidly with high levels of alignment from discontinuous fibres [18]. The elastic modulus, strength, and failure strain of aligned discontinuous fibre reinforced composites (ADFRC) manufactured with the HiPerDiF method have been found to be similar to those of continuous FRP analogues [15].

The HiPerDiF method can also address the sustainability of composite materials in terms of manufacturing and recycling. For example, the production of high performance recycled carbon fibre FRP has been demonstrated by remanufacturing reclaimed fibres with the HiPerDiF method [19,20,21,22,23,24,25]. Lower environmental impact FRP production can be achieved since the method operates with water as the alignment medium, unlike traditional glycerol methods. Despite the use of water, HiPerDiF can be employed to manufacture NF, despite their hydrophilicity, with no significant deleterious effects [23]. Therefore, it is highly likely to obtain a sustainable route for high performance FRP that uses renewable natural fibres in a discontinuous fibre form via the HiPerDiF method.

Building upon a previous study [26] that presents a selection of reinforcement materials for aligned discontinuous natural fibre reinforced polymer composites (ADNFRC), four different types of natural fibres, curaua (Brazilian origin), flax (Polish origin), flax (French origin), and jute (Indian origin), were used in this research work as raw reinforcement materials for the HiPerDiF method to form highly aligned discontinuous fibre preforms. Epoxy, poly(lactic acid) (PLA), and polypropylene (PP) matrices were reinforced with aligned discontinuous natural fibre (ADN) preforms to obtain ADNFRC. ADNFRC were mechanically characterised by using standard tensile test methods. The mechanical and physical properties of the natural fibres were compared within the same and different polymer matrices to reveal their reinforcement performance in ADNFRC. The promising sustainable reinforcement agents for ADNFRC were determined owing to their mechanical performance and physical sufficiency with the production method; hence, the obtained broad results in this study improve the reinforcement selection of materials for high performance and sustainable discontinuous FRP for future studies and industrial applications.

## 2. Materials and Methodology

### 2.1. Fibres

The curaua fibres were collected in Brazil (Santarem, Para) using hand-crafted conventional methods. The jute fibres were Bangla Tossa fibres sourced by Shams UK Ltd.(London, UK), and they were collected in jute cultivation areas in Bangladesh processed by batching, carding, and drawing the filaments. The two flax fibres, sourced from Eco-Technilin-Flaxtape™ (Normandy, Northern France) and flax fibres Ekotex (Namysłów, Poland) were produced via the use of a proprietary process. Since there are two different types of flax fibre, the flax from Poland is referred to as ‘flax-cu’ because it is used for construction (cu), while the flax from France is referred to as ‘flax-ft’ owing to the product name Flaxtape™ (ft). All the fibres were used in the condition they were received. Table 1 demonstrates the properties of the natural fibres.

### 2.2. ADN Preforms

**Figure 1:**
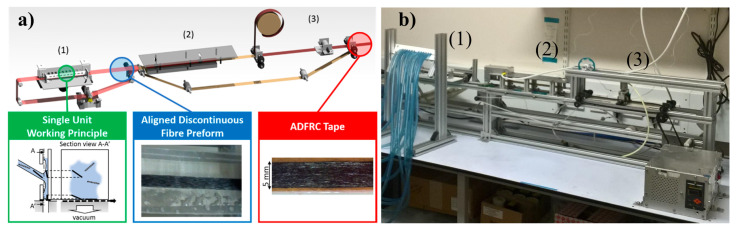
(**a**) The HiPerDiF fibre alignment method principles and dry carbon fibre preform output (stage 2) [23,30], (**b**) an image of the second generation HiPerDiF machine (Bristol Composite Institute, Bristol, UK) highlighted with stages mentioned in Section 2.2.

The HiPerDiF method was used to produce 150 × 5 mm${}^{2}$ ADN preforms that were laid-up to obtain the ADNFRC used in this study. *A schematic of the HiPerDiF discontinuous fibre alignment machine is shown in Figure 1a. Fibres, which can have a length between 1 and 12 mm, were suspended in water, accelerated through a nozzle, and directed in a gap between two parallel plates. The fibre alignment mechanism relies on a sudden momentum change of the fibre-water suspension upon impact with the plate. The fibres then fall on a conveyor stainless mesh belt where the water is removed by suction, stage (1) in Figure 1. The aligned fibre preform was dried with infrared radiation (stage 2) to allow the resin impregnation process* [23], which is the built-in option (stage 3). In this work, stage 3 was not used to impregnate resin to produce ADN prepregs due to the necessity of the hot press for better impregnation and an image of the HiPerDiF machine is shown in Figure 1b.

### 2.3. Matrices and Prepregging

**Figure 2:**
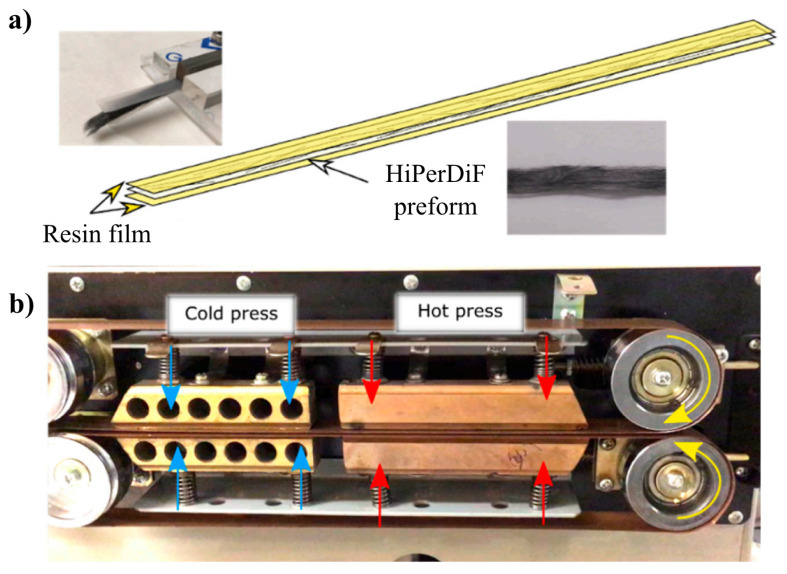
(**a**) An example of the HiPerDiF (carbon fibre) preform sandwiched between two polymeric films before consolidation process (**b**) Prepregging process. Figures were taken from [31].

Three widely available polymers were used as matrices for the ADNFRC; epoxy, PLA, and PP acquired in film form and their properties are shown in Table 1. For an epoxy matrix, Skyflex K51 (SKChemicals, Seoul, Korea) resin films were procured. PLA was sourced from 3D4Makers (Haarlem, The Netherlands) as a 3D printing filament and PLA films were produced by using a 3D printer with a roughly 0.1 mm thickness for the impregnation. PP films were acquired from Propex Fabrics GmbH (Gronau, Germany). For epoxy ADNFRC, a single-ply prepreg was prepared using the consolidation process illustrated in Figure 2 by placing an ADN preform between two epoxy matrix films. A 5 bar pressure was applied for a minute at 60 ${}^{\circ }$C on a slowly moving consolidation belt to obtain an epoxy ADNFRC prepreg. For PLA ADNFRC prepregs, the same process was used but with a different temperature; 210 ${}^{\circ }$C, to achieve better impregnation. To be sure about impregnation of the PP film into the bed of natural fibres, a different method was applied for prepregging. The PP sandwiched ADN preforms were placed in a semi-closed mould and the mould was placed in an oven under vacuum for 3 h at 165 ${}^{\circ }$C. It has been shown that for long term heat treatment, temperatures up to 175 ${}^{\circ }$C may be used without degrading natural fibres [26]. As such, the temperature used in this study, should produce no adverse effects, such as thermal degradation of the fibres.

### 2.4. ADNFRC Manufacture

To manufacture composite specimens four layers of ADN prepregs were stacked in a semi-closed mould and cured by vacuum bag moulding in an autoclave at ∼6.9 bar pressure. The epoxy ADNFRC specimens were cured at 125 ${}^{\circ }$C for 135 min, whereas the PLA and PP ones were cured at 170 ${}^{\circ }$C for 150 min.

### 2.5. Mechanical Testing

**Figure 3:**
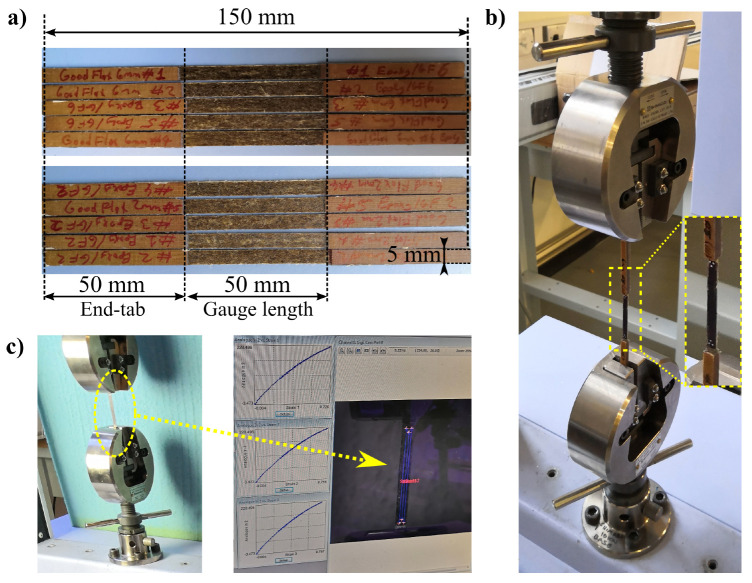
(**a**) Top view of ADNFRC specimens (2 mm and 6 mm flax-ft epoxy composites) and their geometry, (**b**) the tensile test rig with specimen magnified, (**c**) the specimen during measurement and the resulting image on video gauge.

Referring to ASTM D3039/D3039M-17 [32], the ADNFRC specimens were tensile tested on an electro-mechanical testing machine at a test speed of 2 mm/min. The strain was measured by a video extensometer (IMETRUM, Bristol, UK). A 10 kN load cell (Shimadzu, Kyoto, Japan) was used to record the load. The gauge length of the specimens was 50 mm and all specimens were protected against any adverse effects from gripping pressure by attaching glass fibre/epoxy end tabs. Figure 3 shows examples of the top view geometry of ADNFRC and the details of tensile test. As seen in the figure, the nominal ADNFRC specimen sizes for width and length were 5 and 150 mm, respectively; specimen thicknesses where within the approximate ranges 0.39–0.74, 0.14–0.33, 0.36–0.51, and 0.30–0.43 mm for curaua, flax-cu, flax-ft, and jute ADNFRC specimens, respectively.

### 2.6. Visual Characterisation

A high resolution scanner (Epson Expression 11000XL, Epson, Shinjuku, Tokyo) was used to acquire high resolution images of the ADN preforms. An optical microscope (Zeiss Axio Imager M2, Carl Zeiss AG, Oberkochen, Germany) and a scanning electron microscope (Hitachi TM3030Plus, Hitachi, Ltd., Tokyo, Japan) were used to analyse fibre length distributions, cross-sections, and fracture surfaces of the ADNFRC. Cold mounting followed by standard wet grinding and polishing for polymer matrix composites was applied to make the specimens, for cross-section analysis of each ADNFRC under the microscope. The figures of the specimens were shown in Section 3.1 and Section 3.3, and Supplementary Materials.

### 2.7. Density, Porosity, and Water Absorption of ADNFRC

**Figure 4:**
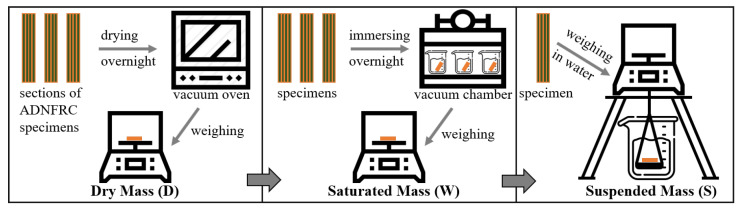
A schematic representation of steps and measurements for density, apparent porosity and water absorption of the composite specimens.

Initially, each ADNFRC specimen was dried overnight, and the dried mass (D) was weighed immediately upon its removal from the vacuum oven used for the drying process. Subsequently, each specimen was immersed in water in a vacuum chamber, again overnight, and the saturated masses (W) of the specimens were weighed in air after its removal. Finally, the suspended weights (S) of specimens were measured while they were immersed in water. Figure 4 shows the experimental procedure schematically. These weights were then used to calculate the apparent density (AD), bulk density (BD), apparent porosity (AP), and water absorption (WA) of the ADNFRC specimens following the ASTM standard C830-00 (Equations (1)–(4), respectively) [33] and the principle of Archimedes (the buoyancy method) using a precision balance (sensitivity 0.1 mg).

(1) $$AD\;\left(g\;{\mathrm{cm}}^{-3}\right)=\frac{D}{D-S}\times \rho_{\mathrm{medium}}$$

(2) $$BD\;\left(g\;{\mathrm{cm}}^{-3}\right)=\frac{D}{W-S}$$

(3) $$AP\;\left(\%\right)=\frac{W-D}{W-S}\times 100$$

(4) $$WA\;\left(\%\right)=\frac{W-D}{D}\times 100$$

where $\rho_{\mathrm{medium}}$ is density of displacement medium.

## 3. Results

### 3.1. Properties of ADN Preforms

**Figure 5:**
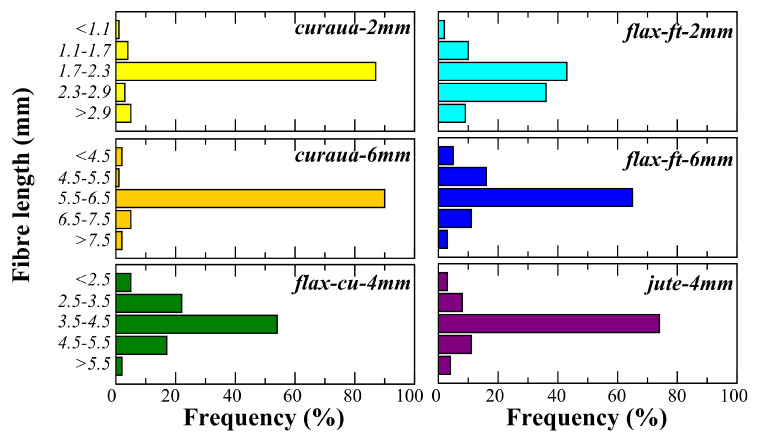
Fibre length distributions of the selected groups of natural fibres.

The investigated length of the discontinuous NF was determined according to previous work on interfacial properties of NF with epoxy resin [26]. For curaua and flax-ft, two different cut lengths were used; (i) the first one was 2 mm, which is close to their critical fibre length ($l_{c}$), which has been shown to be 2.22 and 1.56 mm, respectively, (ii) the second one was 6mm, which is significantly higher than the $l_{c}$. As seen in Table 1, the $l_{c}$ of jute has been shown to be less than 1 mm. Practically the HiPerDiF method works with fibre lengths above 1 mm and below 12 mm. Because of that, the cut length was determined as 4 mm, which is significantly higher than the $l_{c}$ of jute. Flax-cu fibres were sourced already cut from the company as 4 mm. Figure 5 shows the fibre length distribution of NF that are used to produce ADN preforms. The fibre length distributions of NF were obtained by measuring more than 100 fibre lengths in each group type using an optical microscope.

As seen Figure 5, the average length of all groups of NF corresponds to the nominal value with a narrow distribution around it, except flax-ft-2m, which has a wide distribution centered above the nominal value. Curaua fibre type has nearly 90% selected fibre length frequencies for both 2 mm and 6 mm. Likewise, it was found that the selected fibre length frequency is significantly high ∼75% for jute-4mm fibre type and also for flax-ft-6mm, which is ∼65%. The lowest selected fibre length frequency, ∼43%, was seen in flax-ft-2mm.

**Figure 6:**
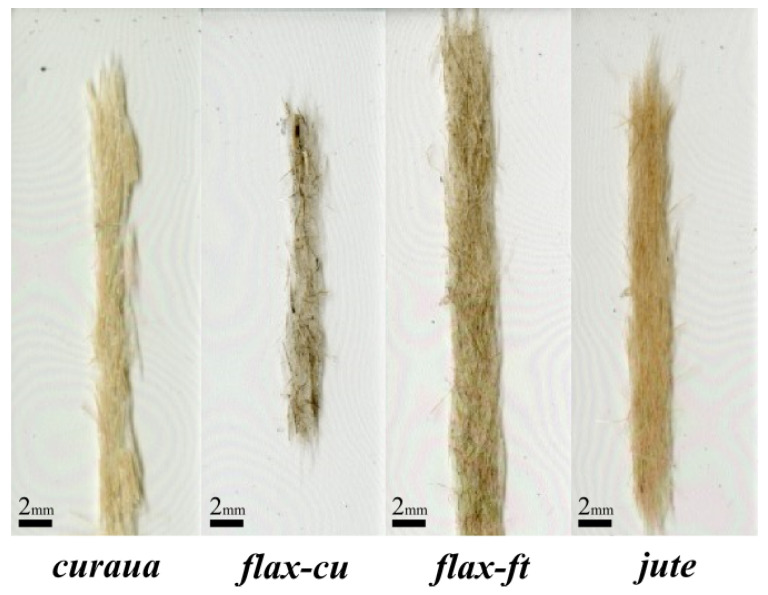
Top view of ADN preforms.

With the discontinuous natural fibres, ADN preforms were produced using the HiPerDiF method. The aerial weight of ADN preforms was determined by dividing the mass of a preform section, measured using a precision balance (sensitivity 0.1 mg), by the area measured using high resolution scans (resolution 3200 dpi) with the ImageJ software. Table 2 shows the aerial weight of ADN preforms and Figure 6 shows the sections of ADN preforms.

The aerial weight of curaua preforms was found be the highest, 116 g/m${}^{2}$, among all other fibre types. Flax-ft and jute preforms have aerial weights of 79 and 65 g/m${}^{2}$, respectively, with a standard deviation ∼6 g/m${}^{2}$. However, it was found that flax-cu preforms exhibit the lowest aerial weight, which is 34 g/m${}^{2}$ with a standard deviation ∼9 g/m${}^{2}$. The reason for these differences is thought to be the considerable amount of debris and the quality of the flax-cu fibre batch sourced from the provider.

### 3.2. Mechanical Properties of ADNFRC

**Figure 7:**
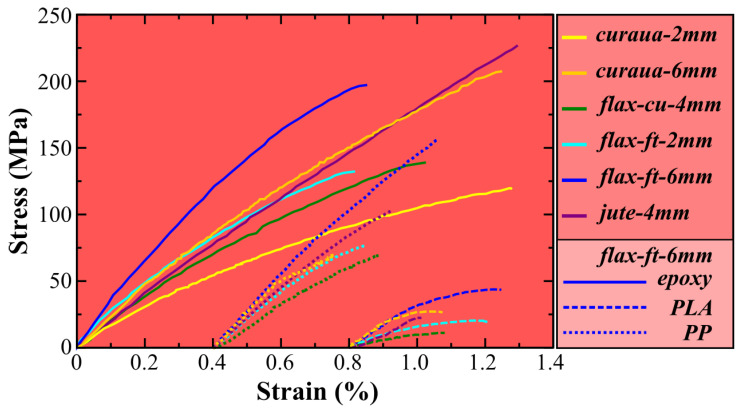
Representative stress–strain curves for the tensile tested aligned discontinuous natural fibre composites specimens and the colour/line code. For PLA and PP curves, 0.8 and 0.4 strain reference points are the starting points, respectively.

Tensile tests were performed to obtain the mechanical properties of epoxy, PLA, and PP ADNFRC. In total, five specimens were tested for each ADNFRC type, except for jute epoxy and flax-cu PP where four specimens were tested. Only one specimen was usable for tensile testing after curing the specimens for flax-cu PLA ADNFRC due to the low packing density of the ADN preform and the low adhesion between PLA and flax-cu. In total, 27 measurements were taken for both the thickness and width of ADNFRC specimens by using a precise micrometer and caliper for converting force to stress. Figure 7 shows the representative stress–strain curves of ADNFRC.

**Figure 8:**
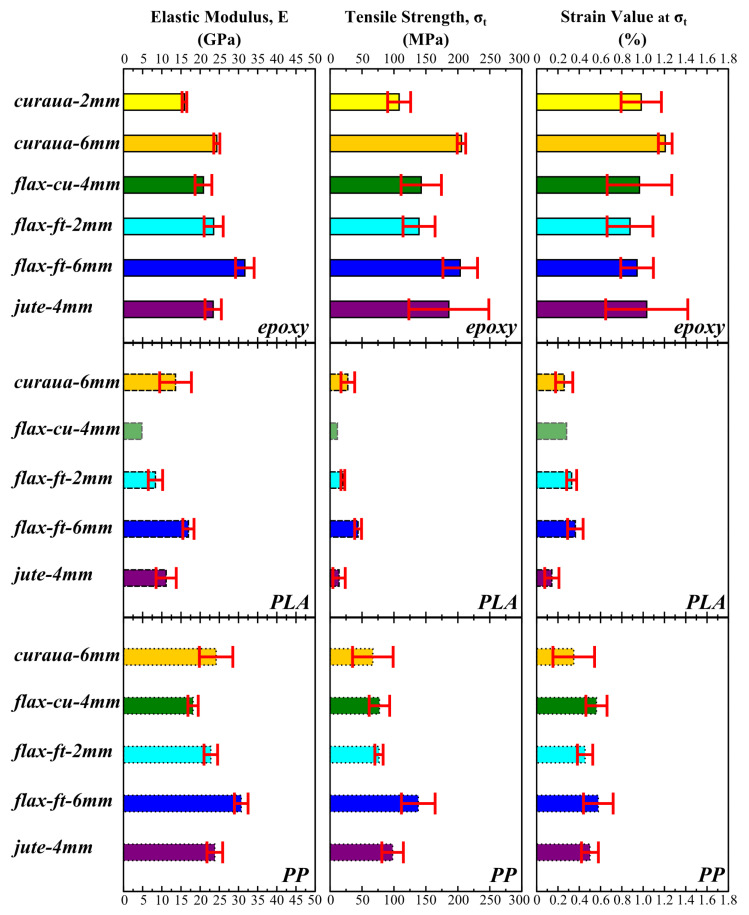
The mechanical properties of aligned discontinuous natural fibre composites, elastic moduli, tensile strength ($\sigma_{t}$) and strain at $\sigma_{t}$, respectively, from left to right. Top to bottom: epoxy (solid line), PLA (dashed line), and PP (dotted line). Error bars indicate standard deviation (SD).

As seen in Figure 7, all PLA ADNFRC exhibit a significant amount of non-linear deformation and they have a smaller linear elastic region compared to epoxy and PP ADNFRC. The general stress–strain behaviour of PP ADNFRC appears to be linear elastic. The linear elastic region of this sample is comparable in strain to epoxy ADNFRC. Most of the epoxy ADNFRC shows what is thought to be non-linear deformation up to fracture. Moreover, it was seen that 2 mm curaua epoxy ADNFRC undergoes non-linear deformation earlier than 6 mm curaua epoxy ADNFRC and shows more non-linear deformation in comparison. Similar trends were observed for 6 mm and 2 mm flax-ft ADNFRC. For epoxy and PLA ADNFRC, 2 mm flax-ft starts showing non-linear deformation earlier than for the 6 mm flax-ft specimen, whereas in PP ADNFRC both of them undergo non-linear deformation at a similar strain. From the stress–strain curves, the elastic modulus of ADNFRC were calculated by calculating the slope of the linear elastic region between 0.05% and 0.015% strain values. Figure 8 shows the elastic moduli, tensile strength ($\sigma_{t}$) and strain at $\sigma_{t}$ of ADNFRC.

The highest elastic modulus values are seen for epoxy ADNFRC, which are between ∼21 and 32 GPa. On the other hand, PLA ADNFRC exhibit the lowest elastic modulus values (between ∼8 and 17 GPa), which are significantly lower compared to epoxy and PP ADNFRC. PP ADNFRC shows similar elastic modulus values (between ∼18 and 31 GPa) compared to those of epoxy ADNFRC. Moreover, the epoxy ADNFRC have the highest $\sigma_{t}$ (between ∼140 and 206 MPa) among all resin types. PLA ADNFRC also show the lowest $\sigma_{t}$ values (<50 MPa), which are lower than those of PP ADNFRC and significantly (statistically significant difference was determined by *t*-test where *p* < 0.01 between all groups) lower than those of epoxy ADNFRC. The PP ADNFRC show moderate $\sigma_{t}$ values (between ∼67 and 138 MPa) and they are significantly lower than those of epoxy ADNFRC. The strain values at $\sigma_{t}$ for epoxy ADNFRC reside between ∼0.9–1.2%. PLA and PP ADNFRC exhibit lower strain values at $\sigma_{t}$ compared to epoxy-based samples, with values residing between ∼0.14–0.36% and ∼0.35–0.58%, respectively.

As seen in Figure 8, 6 mm flax-ft epoxy ADNFRC have the highest elastic modulus values (∼32 GPa) among the four types of fibres followed by 6 mm curaua, 2 mm flax-ft, 4 mm jute, and 4 mm flax-cu epoxy ADNFRC in which the elastic modulus values range between ∼21 and 24 GPa. In total, 2 mm curaua ADNFRC exhibit the lowest elastic modulus values among all epoxy ADNFRC as ∼16 GPa. For $\sigma_{t}$, 6 mm curaua and 6 mm flax-ft epoxy ADNFRC have the highest values, ∼206 and 204 MPa, respectively. Besides, 4 mm jute epoxy ADNFRC exhibit comparably high $\sigma_{t}$, ∼186 MPa with a high standard deviation. The $\sigma_{t}$ values of 4 mm flax-cu and 2 mm flax-ft were found to be ∼143 and 139 MPa, respectively. On the other hand, 2mm curaua epoxy ADNFRC show the lowest $\sigma_{t}$, ∼108 MPa, among all epoxy ADNFRC, similar to the elastic moduli.

Furthermore, 6 mm flax-ft fibre type also exhibits the highest elastic modulus value (∼17 GPa) among all PLA ADNFRC types. In total, 6 mm curaua PLA ADNFRC have the second highest elastic modulus, ∼14 GPa, and 4 mm jute 4-mm PLA ADNFRC the third highest, ∼11 GPa. The lowest elastic modulus values were found for 2 mm flax-ft and 4 mm flax-cu PLA ADNFRC, ∼8 and 5 GPa, respectively. However, since, as explained above, only one sample has been tested, the results for 4 mm flax-cu PLA ADNFRC cannot be considered representative of the material behaviour. The lowest $\sigma_{t}$ value was found for 4 mm flax-cu PLA ADNFRC as ∼12 MPa, however 4 mm jute PLA ADNFRC show low $\sigma_{t}$ value (∼12 MPa) compared to other fibre types with high standard deviation, which indicates it could be much weaker. In total, 6 mm curaua and 2 mm flax-ft PLA ADNFRC have moderate $\sigma_{t}$ compared to other PLA ADNFRC, ∼28 and 20 MPa, respectively. Among all PLA ADNFRC, it was found that 6 mm flax-ft PLA ADNFRC are the strongest (∼44 MPa).

Moreover, 6 mm flax-ft PP ADNFRC show the highest elastic modulus value in composites with the same resin type and also comparable values (∼31 GPa) with epoxy ADNFRC. The same comparable trends are found for 6 mm curaua, 2 mm flax-ft, and 4 mm jute PP ADNFRC where the elastic modulus values reside between ∼23 and 24 GPa. The lowest elastic modulus value was found for 4 mm flax-cu PP ADNFRC as ∼18 GPa, which is lower than that of the epoxy counterpart. In total, 6 mm flax-ft PP ADNFRC was found to be the strongest again within same resin type with a ∼138 MPa tensile strength. The second highest value for $\sigma_{t}$ was observed for the 4 mm jute PP ADNFRC that is close to 100 MPa. In total, 4 mm flax-cu and 2 mm flax-ft exhibit similar $\sigma_{t}$ values-∼77MPa. It was found that the weakest PP ADNFRC is the 6 mm curaua sample—namely a value of ∼67 MPa with a high standard deviation.

### 3.3. Physical Properties of ADNFRC

**Figure 9:**
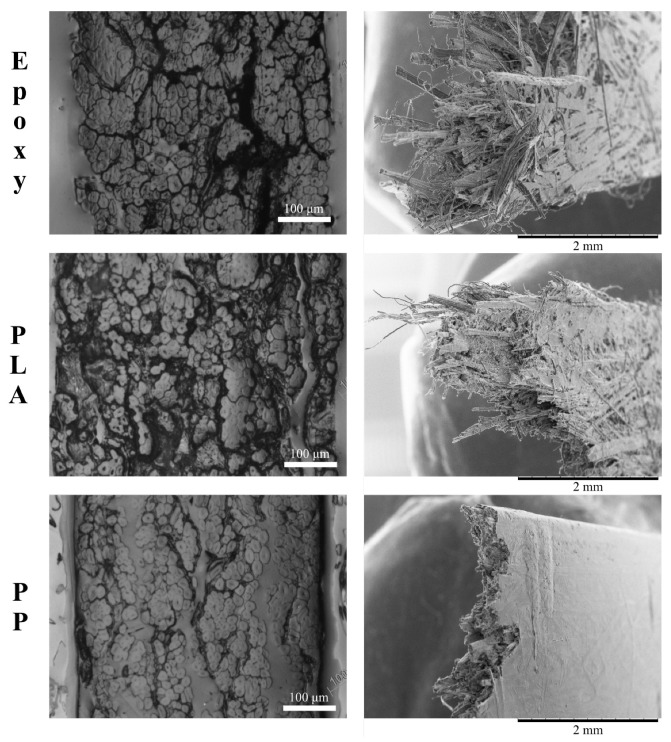
The cross sectional optical images (**left column**) and the fracture area scanning electron micrographs (**right column**) of 6 mm flax-ft aligned discontinuous natural fibre composites.

The cross sections and fracture areas of ADNFRC samples were examined using optical and scanning electron microscopy. Figure 9 shows the cross sectional optical and the fracture area scanning electron micrographs of epoxy, PLA and PP ADNFRC. The 6 mm flax-ft are highlighted, but the micrographs of all ADNFRC are given in the Supplementary Materials (see Figures S1–S5).

The cross-sectional area images shown in Figure 9 (left column) reveal adhesive flaws between the layers of the prepreg, mainly for PLA and epoxy ADNFRC. In contrast, for PP ADNFRC, greater uniformity is noted. In the process of manufacturing PP and PLA ADNFRC, the overlapping of the four prepreg layers is carried out at a temperature of 170 ${}^{\circ }$C. This manufacturing temperature is higher than the melting point of PP (130 ${}^{\circ }$C), which facilitates the reduction of the polymer’s viscosity, providing greater homogenization and adhesion of the layers of the prepreg. However, a temperature of 170 ${}^{\circ }$C is in range of the melting point of PLA (170 ${}^{\circ }$C to 220 ${}^{\circ }$C), giving a viscosity of 10${}^{5}$ cps [34], which is probably the main reason for not obtaining adhesion between the layers. Likewise, the epoxy film during the curing cycle to reach 125 ${}^{\circ }$C present a viscosity between 5000 and 70,000 cps [27], leading to a similar behaviour to the PLA composite during consolidation. Besides, the thickness of the film used affects the conditions for obtaining good adhesion of the layers.

The fracture images shown in Figure 9 on the right reveal that both PLA and epoxy ADNFRC have fibre pull-out, suggesting a low interfacial adhesion between the matrix and the fibres. On the other hand, the images of PP ADNFRC show evidence of a perfect wrapping of PP surrounding the natural fibers that also indicates a good fibre/matrix adhesion. This is evidenced by a clean fracture surface with simultaneous rupture of matrix and the fibres, without the presence of significant pull-out effect. This failure mechanism is inherent to the prepreg manufacturing process, where interfacial bonding between the fibre and the matrix is formed. PP ADNFRC had special treatments where the prepreg was subjected to heating in an oven for 3 h at 165 ${}^{\circ }$C, which probably promoted a better homogeneity of the polymer.

Density, porosity and water absorption are some of the most important properties for FRP in engineering applications. This is especially true for natural FRP due to the adverse effects on their properties and thus their long-term performance [35,36]. The physical properties of 6 mm flax-ft ADNFRC was investigated. Table 3 shows the apparent density, porosity, water absorption, and bulk density of 6 mm flax-ft ADNFRC (F6).

It was seen that F6/Epoxy has the highest density, 1.35 g cm${}^{-3}$, among all F6 ADNFRC. The densities of F6/PLA, and F6/PP were found to be 1.26 and 1.24 g cm${}^{-3}$, respectively. The calculated bulk densities of the fibres are 0.85, 0.78, and 0.99 g cm${}^{-3}$ for F6/epoxy, F6/PLA, and F6/PP, respectively, displaying a different trend compared to apparent densities. Furthermore, it was found that the apparent porosity of F6/epoxy and F6/PLA are nearly twice (∼38%) as high as F6/PP (20%). The same trend is also seen in water absorption values where F6/PP had a 20% water absorption, less than half that of F6/Epoxy and F6/PLA, which are almost similar, 44% and 50%, respectively. It was concluded that the porosity and the water intake of the F6/epoxy and F6/PLA composites significantly higher than F6/PP composites.

By using a simple rule of mixtures approximation, fibre and matrix volume ratio can be roughly estimated for ADNFRC. The density of a composite is determined by the summation of constituent densities weighted according to their volume fractions as shown in the following Equation (5) (neglecting the contribution of voids):

(5) $$\rho_{ADNFRC}=\rho_{NF}\times v_{NF}+\rho_{resin}\times \left(1-v_{NF}\right)$$

where $\rho_{ADNFRC}$, $\rho_{NF}$, and $\rho_{resin}$ are the density of ADNFRC, natural fibre, and polymer, respectively. $v_{NF}$ represents the fibre volume fraction of the composite. Therefore, the density values of F6 ADNFRC (Table 3) were used to calculate $v_{NF}$ of 6 mm flax-ft in ADNFRC by applying Equation (5). Table 4 shows $v_{NF}$ the calculated values for 6 mm flax-ft (F6) fibres in epoxy, PLA, and PP ADNFRC (see Table 1 for the parameters used to calculate $v_{NF}$).

As seen in Table 4, it was calculated that $v_{NF}$ of F6 in PP ADNFRC is the highest fraction among all ADNFRC and more than half, ∼0.52. Figure 9 displays similar high $v_{NF}$ with good adhesion and negligible amount of voids for PP ADNFRC. In addition, the relatively high $v_{NF}$ is also visible for F6/epoxy ADNFRC with the existence of a few voids (Figure 9). For epoxy ADNFRC, $v_{NF}$ of F6 was calculated ∼0.43, which is a favorable value for FRP to achieve high performance as seen in this study. Moreover, it is known that moisture significantly degrades PLA depending on the temperature or pressure [37]. The existence of a significant amount of voids, the presence of the natural fibres, and poor adhesion render PLA ADNFRC degradable in water during the physical property measurements; because of that, the approximation used to calculate the volume fractions cannot be reliable for $v_{NF}$ of F6 in PLA ADNFRC, which was calculated as ∼0.074. Therefore, the anticipated density and $v_{NF}$ for F6/PLA are higher than those obtained in this study. For different fibre types, similar physical properties and trends are foreseen on the grounds that curaua, flax-ft, and jute fibres have similar densities, porosities and water absorption properties [26].

## 4. Discussion

As seen in Figure 6 and Table 2, for flax-ft and jute ADN preforms, an acceptable aerial weight and a good distribution of fibres through the width were obtained, and with these features it is expected to have a high and well distributed fibre volume fraction in ADNFRC, and therefore good mechanical properties. In contrast, those features of curaua or flax-cu ADN preforms were found to be insufficient. One reason for this is thought to be the fibre size (e.g., diameter and thereby aspect ratio) which leads to change of feed rate in the HiPerDiF method. The fibre size for curaua is higher than the other fibre types (as seen Figures S1 and S2), and this affected the process method to produce curaua ADN preform as a slow feed rate, i.e., low water flow to the HiPerDiF alignment head and low conveyor belt speed (Figure 1a), was used to have sufficient aerial weight and better distribution of fibres through the width. Another reason was found in the quality of the source material. There were considerable amounts of debris in the source materials, particularly for flax-cu, but also for curaua in small quantities. The source materials of flax-ft and jute fibres have negligible amounts of impurities.

The alignment of fibres in ADFRC can be examined by analysing fibre orientation using optical microscopy and ellipse fitting of the cross-sections of fibres in ADFRC [31]. However, because of the inherent characteristics of natural fibres, i.e., non-perfectly circular cross-section, their alignment in ADNFRC cannot be determined by using basic characterisation techniques. It is known that the HiPerDiF method, which has a increased fibre alignment compared to other methods, provides 80% and 65% of fibre distribution in the range of $\pm 3^{\circ }$ for a preform and composite form, respectively [17]. Therefore, only a 10% decrease in mechanical performance of ADNFRC is expected due to the fibre misalignment factor, as has been previously demonstrated [15].

A previous study [26] revealed that flax is the stiffest fibre, followed by curaua and jute fibres, respectively. As seen in Figure 8, for fibre lengths higher than the critical fibre length, the stiffness trend of curaua, flax-ft, and jute ADNFRC is in agreement with the intrinsic stiffness trends of the fibres. Furthermore, similar trends can be observed for tensile strength, especially for epoxy ADNFRC, since intrinsically flax and curaua fibres are stronger than jute fibres. However, flax-ft and jute fibres were more compatible with the HiPerDiF method to produce ADN preforms. Therefore, flax-ft ADNFRC show better tensile strength in all polymer ADNFRC compared to the other ADNFRC. Even though curaua fibres are stronger than jute fibres, tensile strength of jute ADNFRC is similar to that of curaua ADNFRC.

The presence of fibres that are shorter than the critical length increases the non-linear behaviour of the ADNFRC. As seen in Figure 7, 2 mm fibre length curaua shows more non-linear behaviour than 6 mm fibre length curaua for epoxy ADNFRC. Similar trend was also observed in 2 mm and 6 mm fibre length flax-ft ADNFRC. In addition, the presence of fibres that are shorter than the critical length reduces the mechanical performance of the ADNFRC drastically. In total, 2 mm curaua epoxy ADNFRC showed the lowest strength and stiffness among those tested, which was expected since critical lengths for curaua have reported to be higher than 2 mm. It was found that there are, respectively, ∼35% and ∼50% reductions in stiffness and tensile strength of curaua epoxy ADNFRC between 2 mm and 6 mm fibre lengths. Since the most of the fibre lengths in the distribution for 2 mm curaua are equal to or less than the critical length (as seen in Figure 5), a significant reduction is seen. Because of the significant amount of mechanical performance loss observed in epoxy ADNFRC and the limited source of curaua fibre, we decided not to proceed with the 2 mm curaua fibre for other polymer ADNFRC. For 2 mm flax-ft, there is less but still a considerable amount of fibre length distribution that is equal to or less than the critical length. Therefore, the noticeable reduction in mechanical properties was expected and observed in all flax-ft ADNFRC. For epoxy ADNFRC, they are ∼25% and ∼32% reductions for stiffness and tensile strength, respectively, between 2 mm and 6 mm flax-ft epoxy ADNFRC. For other flax-ft polymer ADNFRC, it was found that stiffness reduction was ∼52% and ∼25% for PLA and PP ADNFRC, respectively. For tensile strength, the reductions due to fibre length were found to be ∼55% and ∼44% for flax-ft PLA and PP ADNFRC, respectively. Furthermore, the 6 mm flax-ft fibre type showed the best mechanical performance in all polymer ADNFRC compared to other fibre types.

The high homogenization both in the manufacture of PP prepregs and in the consolidation of the final composites are factors that determine the low values of water absorption and porosity of these materials in comparison to others (PLA and epoxy ADNFRC). It is noted from microscopy images (Figure 9), that the PLA and epoxy ADNFRC allow greater water diffusion between the layers due to the lack of full adhesion. Although PP and PLA ADNFRC are manufactured using equal times and temperatures, the PP film benefits from the fact that it can be processed at a lower temperature than PLA. In addition, fibres are limiting factors in the use of high manufacturing temperatures; thus, PLA could not be worked at higher temperature levels, facilitating a greater homogenization of the matrix, as occurred with the PP film.

## 5. Conclusions

In this work, a number of natural fibres (curaua, flax, and jute) were used as sustainable reinforcements to produce high performance aligned discontinuous fibre reinforced polymer (epoxy, PLA, and PP) composites using the HiPerDiF manufacturing method. The mechanical and physical properties of tensile tested ADNFRC were compared to each other within the same and different polymer types. Epoxy ADNFRC and PP ADNFRC were found to be the stiffest materials; however, epoxy ADNFRC are stronger than PP ADNFRC. It was found that PLA ADNFRC shows poor mechanical properties and the reason is considered as the lack of good adhesion and homogenization around the fibres due to limited processing temperature of natural fibres that is less than melting point of PLA. Among all fibre types and fibre lengths, 6 mm flax-ft ADNFRC displayed the best performance in terms of mechanical properties in each ADNFRC polymer group. On the other hand, 6 mm curaua ADNFRC and 4 mm jute ADNFRC showed good mechanical performance either being the second or the third in terms of mechanical properties. Moreover, it was found that PP ADNFRC exhibit significantly lower porosity and water absorption capacity compared to other polymer types and also PP ADNFRC has the lowest density. It was found that the adhesion between PP and the natural fibres is better than epoxy and PLA ADNFRC.

Furthermore, it was found that flax-ft and jute fibres can be processed with the HiPerDiF method since the ADN preforms of flax-ft and jute have acceptable aerial weight and fibre distribution through the width. Jute fibres exhibit good mechanical performance in ADNFRC and suitability with the HiPerDiF method, but flax-ft has also the advantage of having a low environmental impact as a constituent material for ADNFRC because the current market status of the fibres. In addition the location of the production method makes flax fibre more sustainable than other fibre types for the EU and UK [38,39]. In conclusion, flax-ft fibre type was found to be the most promising candidate for these composites. Additionally, this study revealed that 6 mm fibre length for flax is acceptable to achieve high performance in ADNFRC since it is considerably higher than the critical length. On the other hand, jute and curaua fibre types remain as good candidates for future studies when the above mentioned features are satisfied.

The reinforcement phase and the processing method for sustainable high performance ADNFRC were examined. Thanks to simplicity of the method, and the use of a non-chemical medium, the HiPerDiF method already has potential for sustainability whilst being capable of manufacturing high performance composites. In this study, it was demonstrated that NF can be used as a reinforcement material to produce high performance ADFRC with the HiPerDiF method. Therefore, ADNFRC produced with sustainable candidates and manufactured by the HiPerDiF method are promising sustainable composite materials for the future that can be used in most engineering applications, e.g., transport, automobiles, and sporting goods.

## Figures and Tables

**Table 1:** Constituent properties.

Constutient	Density (g cm${}^{-3}$)	Elastic Modulus (GPa)	Tensile Strength (MPa)	Critical Length (mm)	Ref.
Fibres					
curaua	1.50	39	660	2.22	[26]
flax-ft	1.54	52	580	1.56	[26]
flax-cu	1.40	-	-	-	[23]
jute	1.51	27	300	0.84	[26]
Matrices					
epoxy	1.20	-	-	-	[27]
PLA	1.24	-	45	-	[28]
PP	0.92	1.5	-	-	[29]

**Table 2:** Aerial weight of ADN preforms.

ADN Preforms	*Curaua* (2 and 6 mm)	*Flax-cu* (4 mm)	*Flax-ft* (2 and 6 mm)	*Jute* (4 mm)
**Aerial Weight (g/m${}^{2}$)**	116.21	33.53	79.17	65.38
**SD (g/m${}^{2}$)**	5.64	9.09	5.46	6.01

**Table 3:** Physical properties of 6 mm flax-ft aligned discontinuous natural fibre reinforced composites (F6). Errors represent SD.

ADNFRC	Apparent Density (g cm${}^{-3}$)	Apparent Porosity (%)	Water Absorption (%)	Bulk Density (g cm${}^{-3}$)
F6/Epoxy	1.35 ± 0.04	37.15 ± 2.12	44.02 ± 3.43	0.85 ± 0.03
F6/PLA	1.26 ± 0.04	38.52 ± 2.45	49.68 ± 3.48	0.78 ± 0.01
F6/PP	1.24 ± 0.01	20.00 ± 2.89	20.23 ± 3.49	0.99 ± 0.03

**Table 4:** Fibre volume fraction, $v_{NF}$, of 6 mm flax-ft (F6) in the ADNFRC.

ADNFRC	F6/Epoxy	F6/PLA	F6/PP
$v_{NF}$	0.427	Na (0.074 *)	0.516

* the calculated result is not acceptable due to the significant degradation of PLA in water.

## Data Availability

All underlying data are provided in this published article (and its supplementary information files).

## Supplementary Materials

The following are available at https://www.mdpi.com/article/10.3390/ma14081885/s1, Figure S1: The cross sectional optical images (left column) and the fracture area scanning electron micrographs (right column) of 2 mm curaua aligned discontinuous natural fibre composites. Figure S2: The cross sectional optical images (left column) and the fracture area scanning electron micrographs (right column) of 6 mm curaua aligned discontinuous natural fibre composites. Figure S3: The cross sectional optical images (left column) and the fracture area scanning electron micrographs (right column) of 4 mm flax-cu aligned discontinuous natural fibre composites. Figure S4: The cross sectional optical images (left column) and the fracture area scanning electron micrographs (right column) of 2 mm flax-ft aligned discontinuous natural fibre composites. Figure S5: The cross sectional optical images (left column) and the fracture area scanning electron micrographs (right column) of 4 mm jute aligned discontinuous natural fibre composites.
