# Supplementary material for: Natural Fibres as a Sustainable Reinforcement Constituent in Aligned Discontinuous Polymer Composites Produced by the HiPerDiF Method
Source: Materials (Basel). 2021 Apr 10;14(8):1885. doi: 10.3390/ma14081885 (PMC8069299; doi:10.3390/ma14081885)
Supplement: Supplementary file 1 [file materials-14-01885-s001.pdf]

Supporting information to

Natural Fibres as a Sustainable Reinforcement Constituent in Aligned Discontinuous Polymer Composites produced by the HiPerDiF Method

Ali Kandemir[1*], Marco L. Longana[1], Tulio H. Panzera[2], Gilberto G. del Pino[3], Ian Hamerton[1], and Stephen J. Eichhorn[1]

[1]Bristol Composites Institute, Department of Aerospace Engineering, School of Civil, Aerospace, and Mechanical Engineering, University of Bristol, Queen’s Building, University Walk, Bristol, BS8 1TR, UK.

[2]Centre for Innovation and Technology in Composite Materials – CITeC, Department of Mechanical Engineering, Federal University of São João Del Rei - UFSJ, ão João Del-Rei, MG 36307-352, Brazil.

[3]Departamento de Engenharia Mecânica, Universidade do Estado de Amazonas, Manaus-AM, 69050-030, Brazil.

**e-mail address:* ali.kandemir@bristol.ac.uk

The micrographs of the ADNFRC

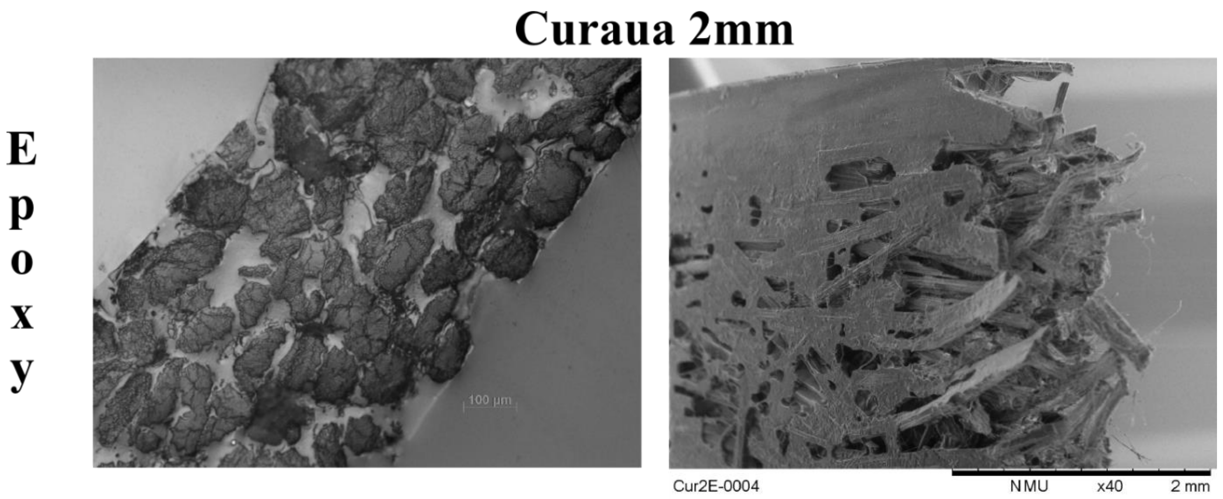

Figure S1. The cross sectional optical images (left column) and the fracture area scanning electron micrographs (right column) of 2 mm curaua aligned discontinuous natural fibre composites.

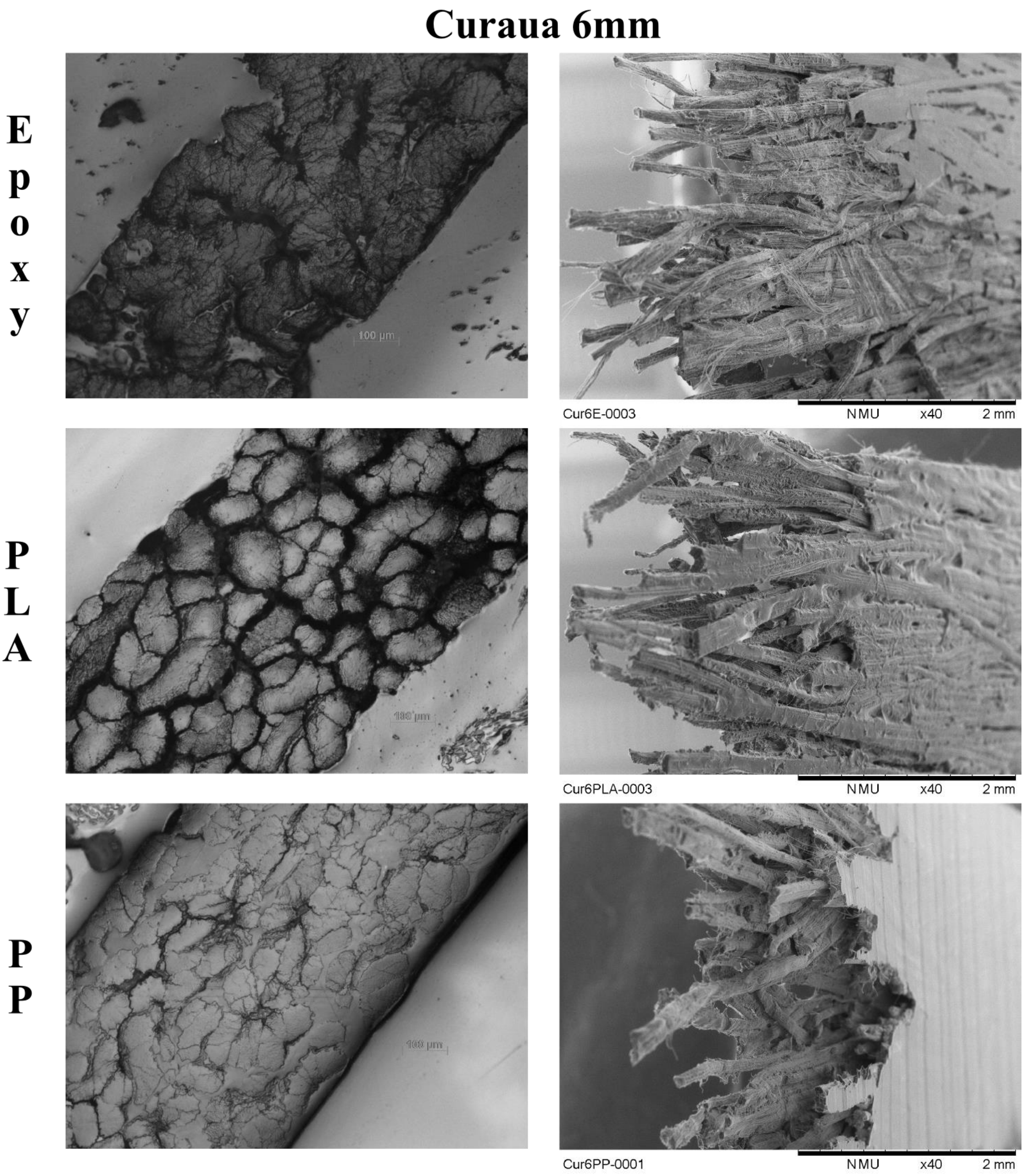

Figure S2. The cross sectional optical images (left column) and the fracture area scanning electron micrographs (right column) of 6 mm curaua aligned discontinuous natural fibre composites.

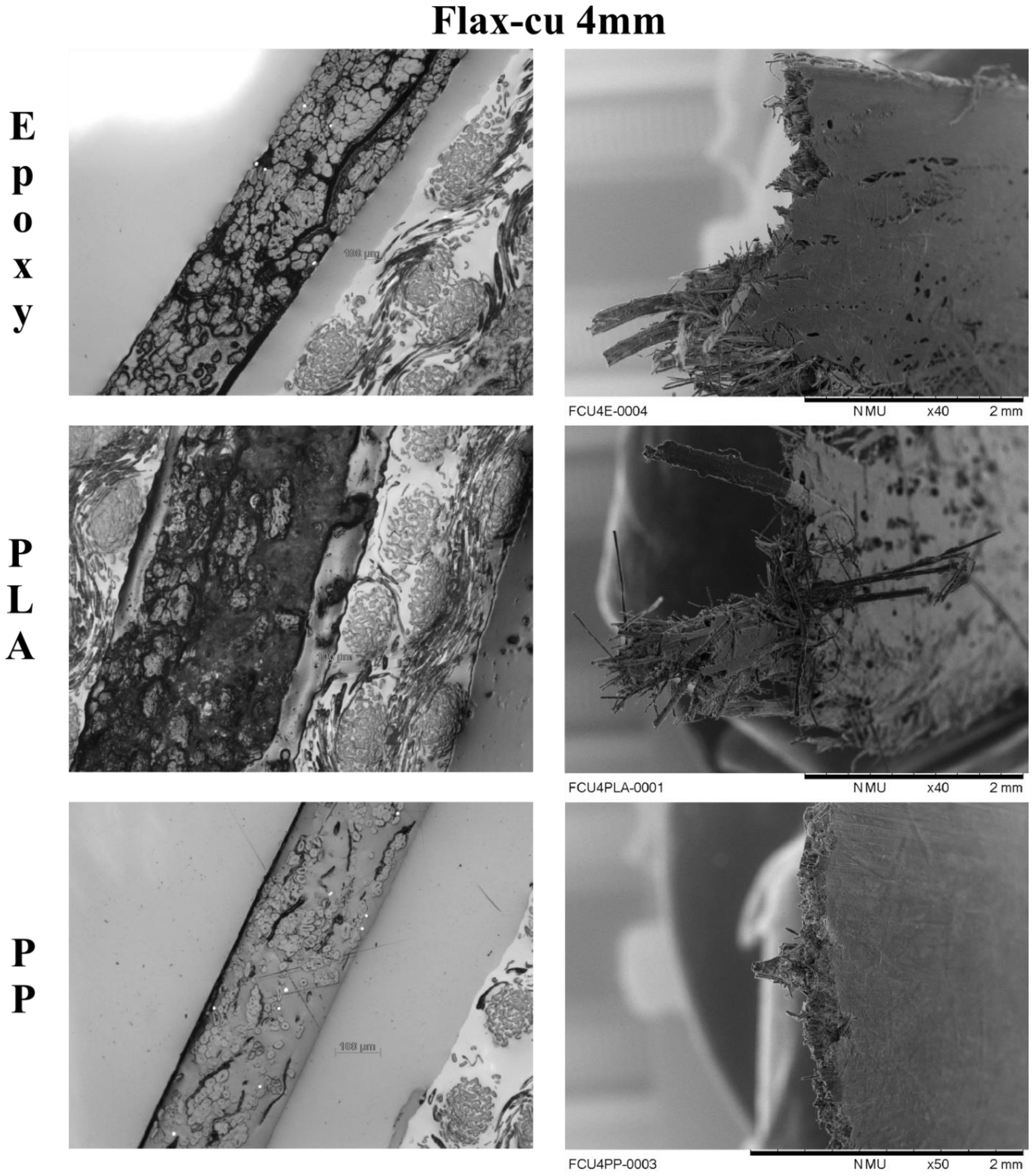

Figure S3. The cross sectional optical images (left column) and the fracture area scanning electron micrographs (right column) of 4 mm flax-cu aligned discontinuous natural fibre composites.

Flax-ft 2mm

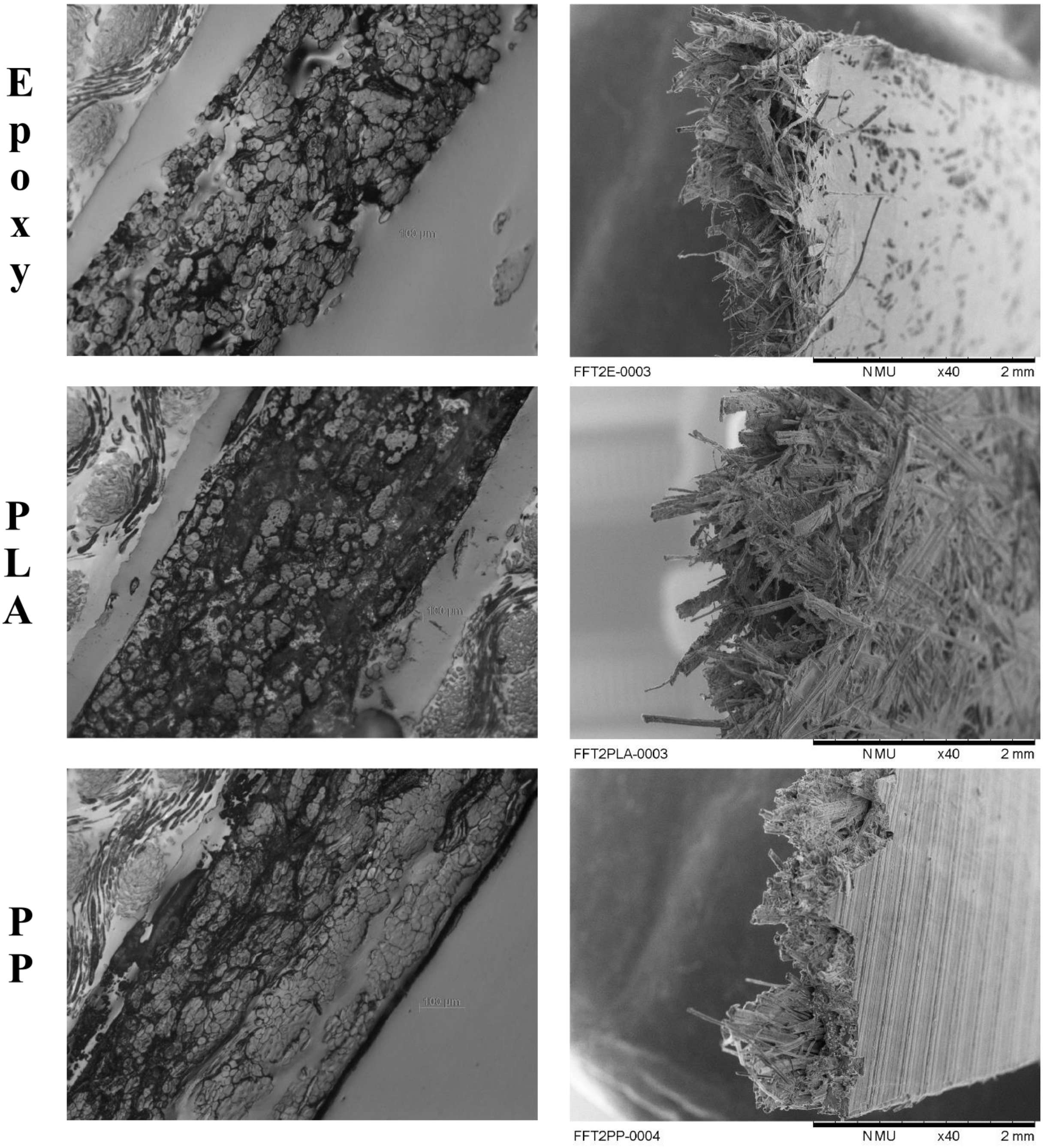

Figure S4. The cross sectional optical images (left column) and the fracture area scanning electron micrographs (right column) of 2 mm flax-ft aligned discontinuous natural fibre composites.

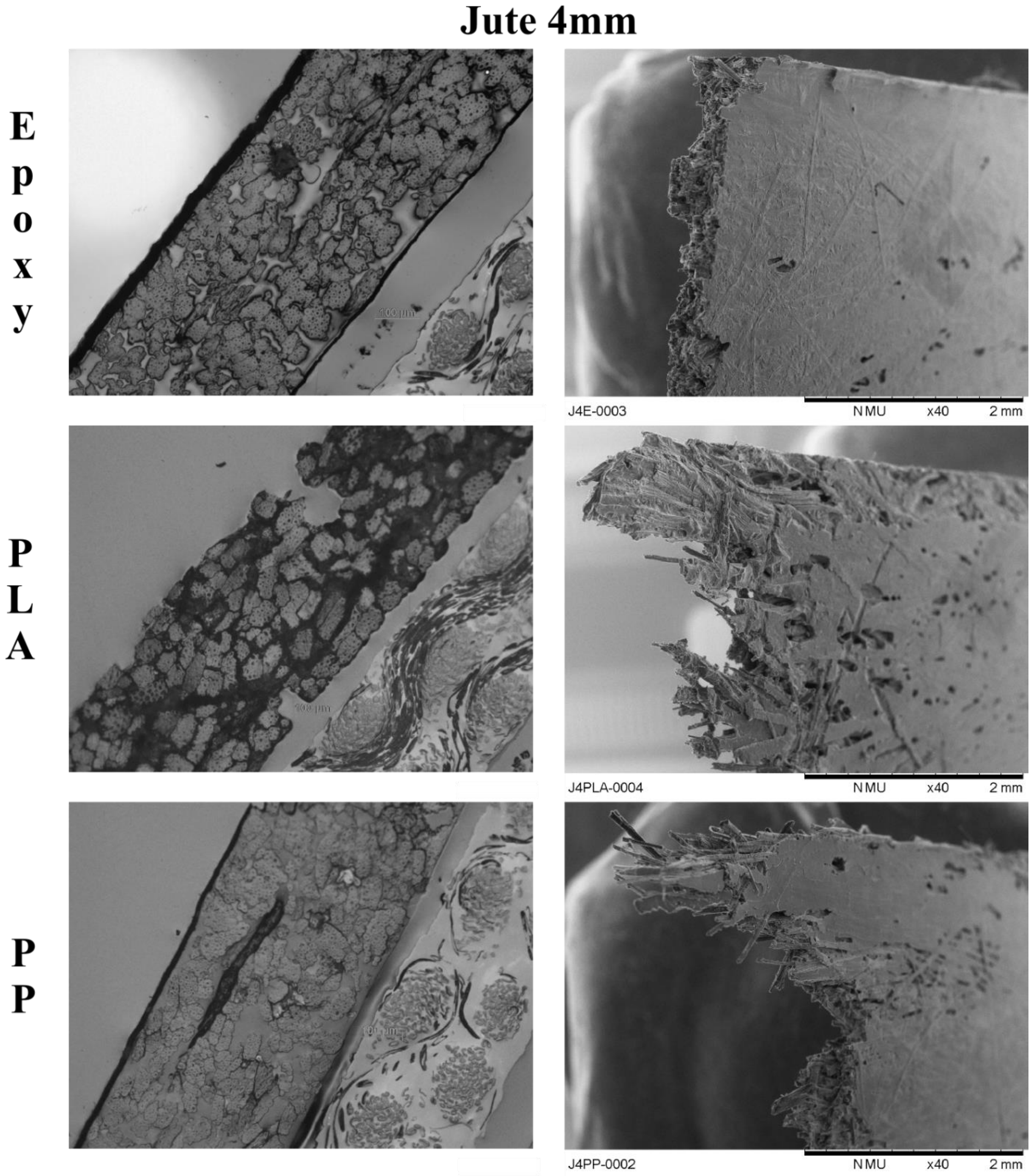

Figure S5. The cross sectional optical images (left column) and the fracture area scanning electron micrographs (right column) of 4 mm jute aligned discontinuous natural fibre composites.
